# Supplementary material for: Lentiviral vector induces high-quality memory T cells via dendritic cells transduction
Source: Commun Biol. 2021 Jun 10;4:713. doi: 10.1038/s42003-021-02251-6 (PMC8192903; doi:10.1038/s42003-021-02251-6)
Supplement: Supplementary file 2 — Supplementary Information [file 42003_2021_2251_MOESM2_ESM.pdf]

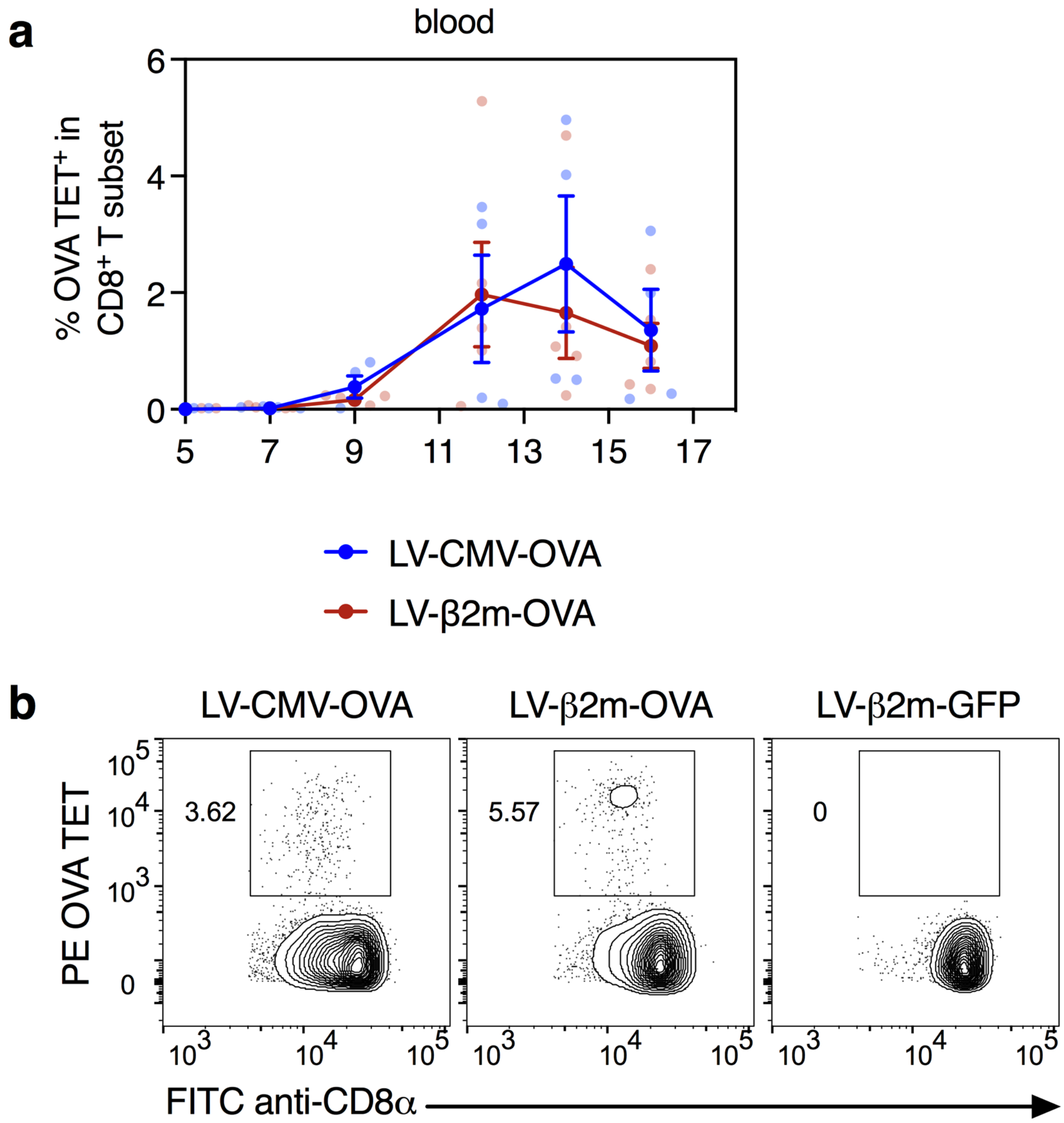

Supplementary Figure 1. Frequency of OVA-specific CD8$^+$ T cells after i.v. immunization with lentiviral vector

(a) C57BL/6 mice (n = 4/group for LV-CMV and n = 5/group for LV-β2m) were immunized i.v. with 5 × 10^7 TU of LV-CMV or LV-β2m encoding for OVA:242-353 immunodominant, H-2K^b-restricted epitope. The frequencies of OVA-specific CD8$^+$ T cells in the blood were determined by the use of a PE-conjugated (K^b-SIINFEKL)$_4$ tetramer (PE OVA TET) by cytometry at 5, 7, 9, 12, 14 and 16 dpi, expressed as mean ± SEM. Smaller dots represent biological replicates. **(b)** Representative cytometric plots for OVA tetramer CD8$^+$ T cells at 14 dpi, with LV-β2m-GFP-immunized mice used as negative controls.

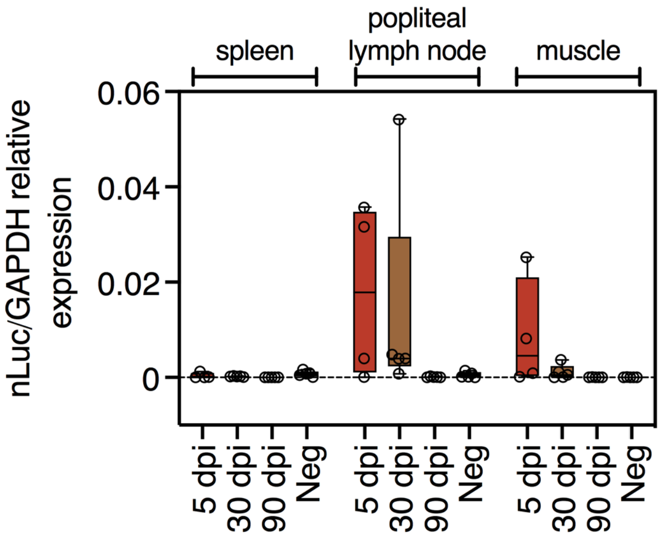

Supplementary Figure 2. Transgene persistence after i.m. immunization with lentiviral vector

C57BL/6 mice (n = 4 for 5 dpi positive control, n = 5 for 30 dpi, 90 dpi and negative control) were immunized i.m with 5 × 10^7 TU of LV-β2m-nLuc. Spleen, popliteal lymph node and muscle were collected at 30 or 90 dpi for detection of nLuc transgene via quantitative PCR (qPCR). Organs of immunized mice were also collected at 5 dpi as positive control. LV-β2m-GFP-immunized mice were used as negative control. Results are shown in box and whiskers plot as ratio of nLuc/GAPDH signal. The medians are marked by horizontal line inside the box and the end of the box represents the interquartile range. The horizontal lines outside the box represent the maximum and minimum values. Circles represent biological replicates.

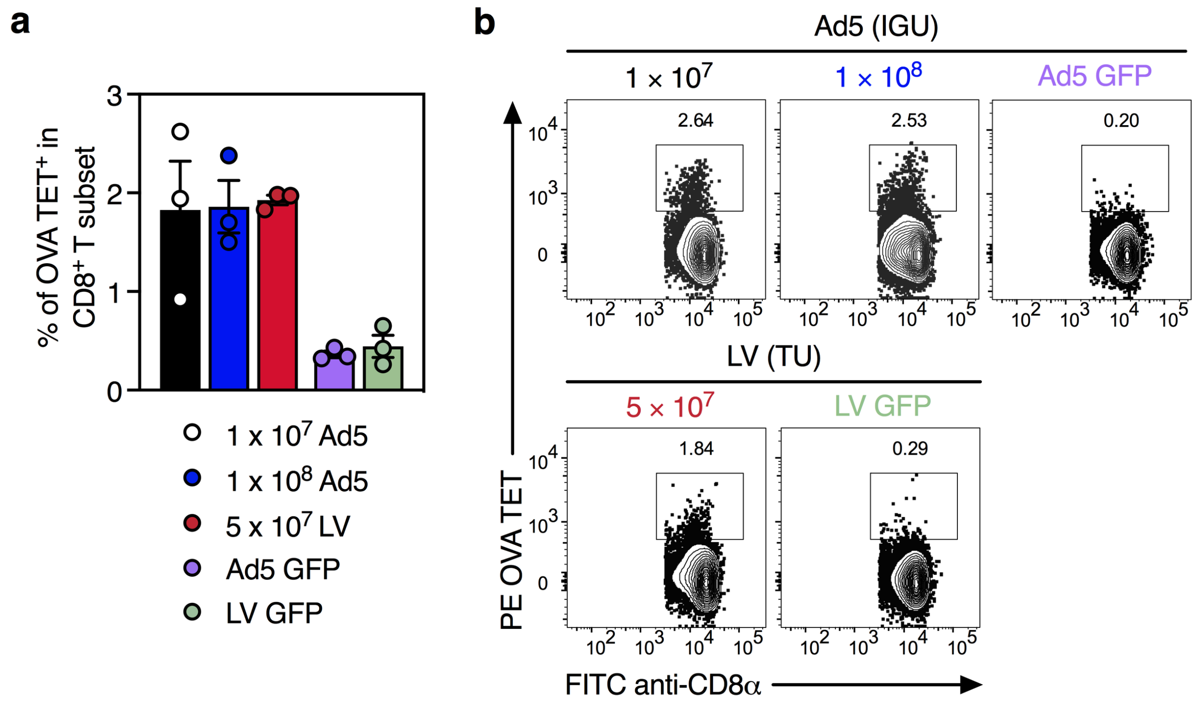

Supplementary Figure 3. Optimal immunization dose of Ad5-CMV vector. (a) C57BL/6 mice (n = 3/group) were immunized i.m with the indicated doses of Ad5-CMV or LV-β2m coding for OVA:242-353 immunodominant, H-2K^b-restricted epitope. The frequencies of OVA-specific CD8$^+$ T cells in the spleen were determined by the use of a PE-conjugated (K^b-SIINFEKL)$_4$ tetramer (PE OVA TET) by cytometry at 28 dpi. Horizontal bars represent mean ± SEM. Each circle represents biological replicate. **(b)** Cytometric plots for a representative positive mouse and a negative control are shown.

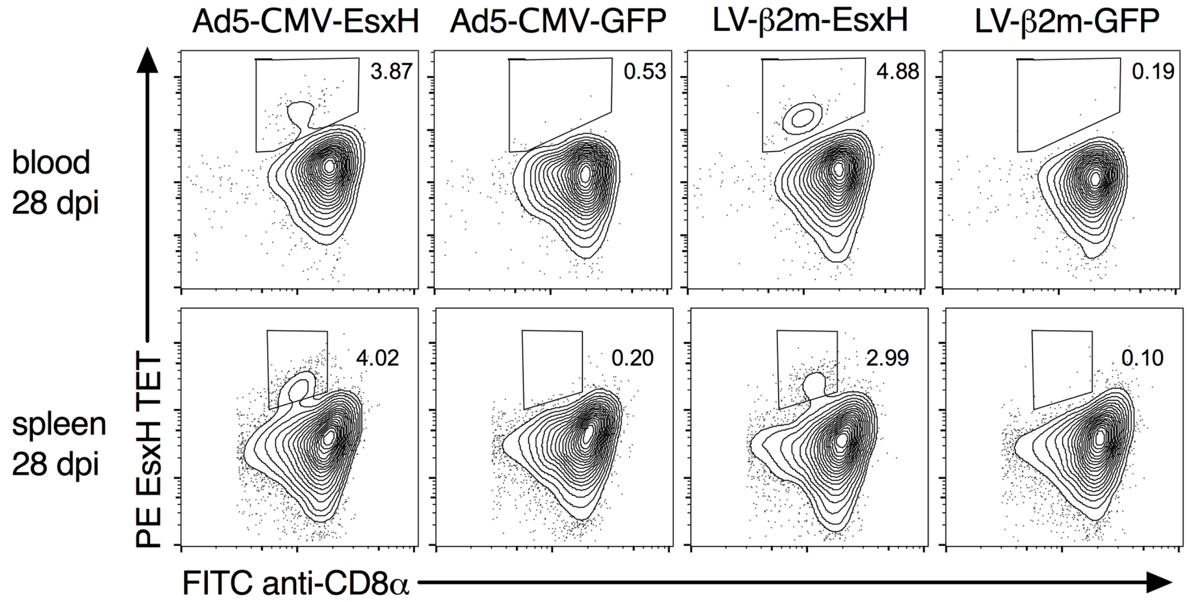

Supplementary Figure 4. Representative cytometric plots for EsxH tetramer CD8$^+$ T-cells induced after immunization with Ad5 or . C57BL/6 mice immunized i.m with 1 × 10^7 IGU of Ad5-CMV-EsxH or 5 × 10^7 TU of LV-β2m-EsxH, while negative control mice were injected with Ad5-CMV-GFP or LV-β2m-GFP. EsxH-specific CD8$^+$ T cells were detected in the blood and spleen by use of PE-(K^b-IMYNYPAM)$_4$ tetramer (PE EsxH TET) by cytometry at 28 dpi.

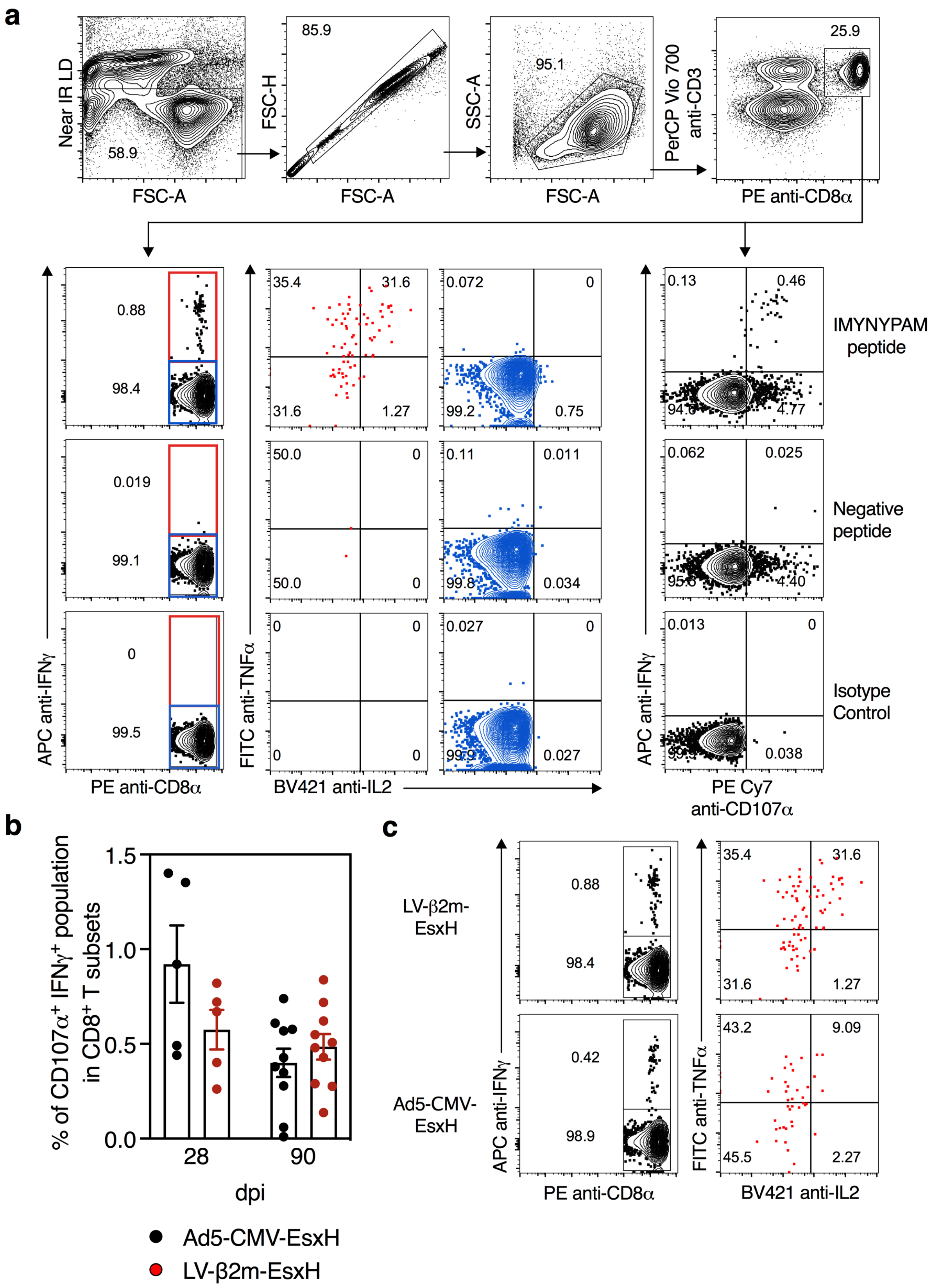
a
Near IR LD
FSC-A
58.9
FSC-H
FSC-A
85.9
SSC-A
FSC-A
95.1
PerCP Vio 700 anti-CD3
PE anti-CD8α
25.9
APC anti-IFNγ
PE anti-CD8α
0.88
98.4
0.019
99.1
0
99.5
FITC anti-TNFα
BV421 anti-IL2
35.4
31.6
31.6
1.27
0.072
0
99.2
0.75
50.0
0
50.0
0
0.11
0.011
99.8
0.034
0
0
0
0
0.027
0
99.9
0.027
APC anti-IFNγ
PE Cy7 anti-CD107α
0.13
0.46
94.6
4.77
0.062
0.025
95.6
4.40
0.013
0
99.9
0.038
IMYNYPAM peptide
Negative peptide
Isotype Control
b
% of CD107α+ IFNγ+ population in CD8+ T subsets
1.5
1.0
0.5
0.0
28
90
dpi
Ad5-CMV-EsxH
LV-β2m-EsxH
c
LV-β2m-EsxH
Ad5-CMV-EsxH
APC anti-IFNγ
PE anti-CD8α
0.88
98.4
0.42
98.9
FITC anti-TNFα
BV421 anti-IL2
35.4
31.6
31.6
1.27
43.2
9.09
45.5
2.27

Supplementary Figure 5. Gating strategy to define diverse functional subsets of antigen-specific CD8$^+$ T cells

C57BL/6 mice (n = 5/group for 28 dpi and n = 10/group for 90 dpi) were immunized i.m with 1 × 10^7 IGU of Ad5-CMV-EsxH or 5 × 10^7 TU of LV-β2m-EsxH, while negative control mice were injected with Ad5-CMV-GFP or LV-β2m-GFP. Cytometric analyses of antigen-specific T-cell response were performed at 28 or 90 dpi. **(a)** Representative ICS data of splenocytes after stimulation with EsxH:3-11 peptide, negative control peptide and isotype control. **(b)** Percentage of CD8$^+$ CD107α$^+$ IFNγ$^+$ splenocytes at 28 and 90 dpi. Horizontal bars represent means ± SEM. Each dot represents biological replicate. **(c)** Representative ICS data of triple positive splenocytes from Ad5-CMV-EsxH- or LV-β2m-EsxH-immunized mice.
