# Supplementary material for: Lentiviral vector induces high-quality memory T cells via dendritic cells transduction
Source: Commun Biol. 2021 Jun 10;4:713. doi: 10.1038/s42003-021-02251-6 (PMC8192903; doi:10.1038/s42003-021-02251-6)
Supplement: Supplementary file 3 — Description of Supplementary Files [file 42003_2021_2251_MOESM3_ESM.pdf]

**Description of Additional Supplementary Files**

**File name:** Supplementary Data 1

**Description:** All source data underlying the graphs presented in the main figures in Excel format.
