# Supplementary material for: Lentiviral vector induces high-quality memory T cells via dendritic cells transduction
Source: Commun Biol. 2021 Jun 10;4:713. doi: 10.1038/s42003-021-02251-6 (PMC8192903; doi:10.1038/s42003-021-02251-6)
Supplement: Supplementary file 5 — Reporting Summary [file 42003_2021_2251_MOESM5_ESM.pdf]

nature research

Corresponding author(s): Laleh MAJLESSI
Last updated by author(s): May 14, 2021

Reporting Summary

Nature Research wishes to improve the reproducibility of the work that we publish. This form provides structure for consistency and transparency in reporting. For further information on Nature Research policies, see our Editorial Policies and the Editorial Policy Checklist.

Statistics

For all statistical analyses, confirm that the following items are present in the figure legend, table legend, main text, or Methods section.

n/a	Confirmed	
☐	☒	The exact sample size (*n*) for each experimental group/condition, given as a discrete number and unit of measurement
☐	☒	A statement on whether measurements were taken from distinct samples or whether the same sample was measured repeatedly
☐	☒	The statistical test(s) used AND whether they are one- or two-sided<br>*Only common tests should be described solely by name; describe more complex techniques in the Methods section.*
☒	☐	A description of all covariates tested
☒	☐	A description of any assumptions or corrections, such as tests of normality and adjustment for multiple comparisons
☐	☒	A full description of the statistical parameters including central tendency (e.g. means) or other basic estimates (e.g. regression coefficient) AND variation (e.g. standard deviation) or associated estimates of uncertainty (e.g. confidence intervals)
☒	☐	For null hypothesis testing, the test statistic (e.g. *F*, *t*, *r*) with confidence intervals, effect sizes, degrees of freedom and *P* value noted<br>*Give P values as exact values whenever suitable.*
☒	☐	For Bayesian analysis, information on the choice of priors and Markov chain Monte Carlo settings
☒	☐	For hierarchical and complex designs, identification of the appropriate level for tests and full reporting of outcomes
☒	☐	Estimates of effect sizes (e.g. Cohen's *d*, Pearson's *r*), indicating how they were calculated

Our web collection on statistics for biologists contains articles on many of the points above.

Software and code

Policy information about availability of computer code

Data collection	In vivo bioluminescene images were imaged using the Living Image Software. Flow cytometry samples were acquired using Attune NxT Software version 4.2.
Data analysis	In vivo bioluminescene images were analyzed using the Living Image Software. Flow cytometry data were analyzed using FlowJo Software. qPCR data were analyzed with Microsoft Excel 2016. Graph and statistical analysis were performed using GraphPad Prism (version 8). Figures were prepared in GraphPad Prism (version 8)

For manuscripts utilizing custom algorithms or software that are central to the research but not yet described in published literature, software must be made available to editors and reviewers. We strongly encourage code deposition in a community repository (e.g. GitHub). See the Nature Research guidelines for submitting code & software for further information.

Data

Policy information about availability of data

All manuscripts must include a data availability statement. This statement should provide the following information, where applicable:

- Accession codes, unique identifiers, or web links for publicly available datasets
- A list of figures that have associated raw data
- A description of any restrictions on data availability

The authors declare that all data supporting the findings are available within the article and the supplementary figures. The source data for main and supplementary figures are provided as Supplementary Data 1. All other data are available upon reasonable request from the corresponding authors.

Field-specific reporting

Please select the one below that is the best fit for your research. If you are not sure, read the appropriate sections before making your selection.

- [x] Life sciences
- [ ] Behavioural & social sciences
- [ ] Ecological, evolutionary & environmental sciences

For a reference copy of the document with all sections, see nature.com/documents/nr-reporting-summary-flat.pdf

Life sciences study design

All studies must disclose on these points even when the disclosure is negative.

Sample size	For in vivo bioluminescence imaging experiment, in vivo immune cell tropism study, comparison of T-cell responses between LV-CMV and LV-β2m and therapeutic immunization of EG.7, 3-6 mice per experimental condition were used; the number of mice in these experiments were determined based on previous published work and preliminary studies.<br><br>5-10 mice were used for the side-by-side comparison study of LV and Ad5. The effect size for this study was calculated using G*power with α err prob=0.05 and power=0.8.
Data exclusions	No data are excluded from the analyses
Replication	Each experiments reported in the manuscript was replicated in multiple animals (between 3 to 10 per experimental condition). Ex vivo-based assay performed in 2 - 3 independent experiments and were replicated successfully.
Randomization	Animals were randomly allocated to experimental and control groups. Cell lines from different passage were utilized for experiments.
Blinding	Investigators were not blinded in during data collection because all samples were processed using standard protocol that should not bias experimental outcomes. Blinding during analysis was also no required as the differences between different experimental groups were visually apparent.

Reporting for specific materials, systems and methods

We require information from authors about some types of materials, experimental systems and methods used in many studies. Here, indicate whether each material, system or method listed is relevant to your study. If you are not sure if a list item applies to your research, read the appropriate section before selecting a response.

Materials & experimental systems

n/a	Involved in the study	
☐	☒	Antibodies
☐	☒	Eukaryotic cell lines
☒	☐	Palaeontology and archaeology
☐	☒	Animals and other organisms
☒	☐	Human research participants
☒	☐	Clinical data
☒	☐	Dual use research of concern

Methods

n/a	Involved in the study	
☒	☐	ChIP-seq
☐	☒	Flow cytometry
☒	☐	MRI-based neuroimaging

Antibodies

Antibodies used	Antibodies used in this study have been included in the materials and methods section of this manuscript
Validation	Antibodies are validated by manufacturer

Eukaryotic cell lines

Policy information about cell lines

Cell line source(s)	The human embryonic kidney 293 (HEK-293) T cells were obtained from ATCC. The 293A cells were obtained from ThermoFisher as part of the ViraPower™ Adenoviral Expression System Kit.
Authentication	Cell lines were authenticated by the supplier

Mycoplasma contamination	293T cell line was tested negative of mycoplasm contamination using MycoAlert Detection Kit (Lonza). 293A cell line was not tested for mycoplasma contamination
Commonly misidentified lines (See ICLAC register)	No commonly misidentified cell lines were used in this study

Animals and other organisms

Policy information about studies involving animals; ARRIVE guidelines recommended for reporting animal research

Laboratory animals	- Female, 5 - 6-week-old C57BL/6JRj (H-2b) mice from Janvier Laboratory (Saint-Berthevin, France) and used at 8-12 week old<br>- Female CD Sprague-Dawley rats (201-225 grams) from Charles River Laboratories (Saint Germain Nuelles, France)
Wild animals	n/a
Field-collected samples	n/a
Ethics oversight	All studies were performed after approval by Institut Pasteur Safety, Animal Care and Use Committee, under local ethical committee protocol agreement (APAFIS#19863-2019031917064954, APAFIS#20289-2019060616332025 and APAFIS #20981-2019060616411273) and after approval by the Ethical Commission of Sciensano (ethical committee project agreement 20180910-01).

Note that full information on the approval of the study protocol must also be provided in the manuscript.

Flow Cytometry

Plots

Confirm that:

- [x] The axis labels state the marker and fluorochrome used (e.g. CD4-FITC).
- [x] The axis scales are clearly visible. Include numbers along axes only for bottom left plot of group (a 'group' is an analysis of identical markers).
- [x] All plots are contour plots with outliers or pseudocolor plots.
- [x] A numerical value for number of cells or percentage (with statistics) is provided.

Methodology

Sample preparation	The detailed sample preparation are described in the materials and methods section.
Instrument	Attune NxT Flow Cytometer, ThermoFisher Scientific
Software	Flow cytometry samples were acquired using Attune NxT Software version 4.2. Flow cytometry data were analyzed using FlowJo Software.
Cell population abundance	The abundance of cells post-sort was dependent upon cell type. Due to the low abundance of CD11c+ cells in lymph node, CD11c+ cells were first enriched using magnetic purification and further sorted into several CD11c+ cell types. The purify of CD8α+ cells after magnetic purification was confirmed by flow cytometry to be more than 90 %.
Gating strategy	Cells were gated on the basis of FSC-A/SSC-A to identify the location of lymphocytes. Unstained cells, single stained cells or negative controls were used to established gates for each populations. Gating strategy for the different immune panels are shown clearly in the manuscript.

- [x] Tick this box to confirm that a figure exemplifying the gating strategy is provided in the Supplementary Information.
